# Implicit and Explicit Gender Beliefs in Spatial Ability: Stronger Stereotyping in Boys than Girls

**DOI:** 10.3389/fpsyg.2016.01114

**Published:** 2016-07-26

**Authors:** Karin M. Vander Heyden, Nienke M. van Atteveldt, Mariette Huizinga, Jelle Jolles

**Affiliations:** Department of Educational Neuroscience, LEARN! Research Institute for Learning and Education, Vrije UniversiteitAmsterdam, Netherlands

**Keywords:** gender stereotypes, gender beliefs, spatial ability, sex differences, children

## Abstract

Sex differences in spatial ability are a seriously debated topic, given the importance of spatial ability for success in the fields of science, technology, engineering, and mathematics (STEM) and girls' underrepresentation in these domains. In the current study we investigated the presence of stereotypic gender beliefs on spatial ability (i.e., “spatial ability is for boys”) in 10- and 12-year-old children. We used both an explicit measure (i.e., a self-report questionnaire) and an implicit measure (i.e., a child IAT). Results of the explicit measure showed that both sexes associated spatial ability with boys, with boys holding more male stereotyped attitudes than girls. On the implicit measure, boys associated spatial ability with boys, while girls were gender-neutral. In addition, we examined the effects of gender beliefs on spatial performance, by experimentally activating gender beliefs within a pretest—instruction—posttest design. We compared three types of instruction: boys are better, girls are better, and no sex differences. No effects of these gender belief instructions were found on children's spatial test performance (i.e., mental rotation and paper folding). The finding that children of this age already have stereotypic beliefs about the spatial capacities of their own sex is important, as these beliefs may influence children's choices for spatial leisure activities and educational tracks in the STEM domain.

“Is Spatial Ability a Boy Thing?

Children's Beliefs about Sex Differences in the Spatial Domain and the Effects on Spatial Performance.”

## Introduction

Spatial reasoning is an important and unique aspect of children's thinking, consisting of different abilities involving the mental representation and manipulation of spatial information (e.g., object rotation, mental folding, perspective taking, and navigating). Children's spatial abilities are related to many of their daily activities, such as finding their way to school, sports, and playing games (Newcombe and Frick, 2010). Importantly, children's spatial abilities are fundamental to quantitative reasoning, such as mathematics (e.g., Nuttall et al., 2005; Newcombe et al., 2015), and to later success and achievement in the domain of science, technology, engineering, and mathematics (STEM; Shea et al., 2001; Wai et al., 2009). Given the underrepresentation of girls in these domains, the development of sex differences in spatial ability is an important topic of investigation.

There is growing consensus that sex differences in spatial ability emerge in the course of childhood, notably around 10-years of age (e.g., Johnson and Meade, 1987; Titze et al., 2010a; Neuburger et al., 2011; Hoyek et al., 2012). Better performance of boys is particularly evident for mental rotation tasks. On other spatial tasks, such as the Paper Folding Test and WISC Block Design test, no consistent sex differences are found (Voyer et al., 1995). This relatively late emergence of sex differences in mental rotation suggests that socio-psychological and experiential factors play an important role in their onset. There is strong evidence that sex differences in spatial ability are related to sex differences in spatial experience, such as play with spatial toys and participation in spatial school subjects (e.g., Baenninger and Newcombe, 1995; Jirout and Newcombe, 2015; Moè, 2016). Another relevant factor is stereotyping. Spatial ability is stereotypically considered a male aptitude (Nash, 1979; Neuburger et al., 2013). In the long term, stereotypic beliefs about the masculinity of spatial ability may lead to sex differences in spatial achievement by affecting boys' and girls' self-concept of ability and by influencing the degree to which they are involved in relevant spatial activities at home and at school (e.g., Baenninger and Newcombe, 1995; Bussey and Bandura, 1999; Dweck, 1999; Jirout and Newcombe, 2015). In the short term, the activation of positive or negative beliefs about the ability of one of both sexes may lead to sex differences in spatial performance in a test situation.

Especially in late childhood, stereotypic gender beliefs may provide an explanation for the observed sex differences in spatial ability. Around 10 years of age children have developed awareness of stereotypes (McKown and Weinstein, 2003) and make important steps in the development of self-concepts of ability (Berk, 2013). However, the literature is inconclusive whether at this age (1) children (already) have stereotypic beliefs on sex differences in spatial ability; (2) there are short-term effects of gender beliefs on spatial test performance, as observed in studies with adults (e.g., Moè and Pazzaglia, 2006; Heil et al., 2012). We investigated these two topics in 10- and 12-year-old children, by examining with different types of measures the presence of stereotypic gender beliefs on spatial ability and by examining the effects of experimentally activating beliefs about sex differences in spatial ability (i.e., instructing children that either boys are better, girls are better, or that there are no sex differences) on spatial performance.

### Stereotypic gender beliefs on spatial ability in children

Stereotypical beliefs about certain abilities or activities being typical for one of both sexes (e.g., the belief that spatial ability is for boys) can be assessed with either explicit or implicit measures. Explicit measures are for example self-report questionnaires asking children whether they consider certain abilities and/or activities as more boyish or more girlish (e.g., Neuburger et al., 2015). Implicit measurement methods on the other hand are indirect, in that participants are not aware on which concepts or on which relations between concepts they are reporting. An example is the Implicit Association Test (IAT, Greenwald et al., 1998), a computer task requiring participants to categorize as quickly as possible words from different concepts (e.g., boys' and girls' names and words related to different school subjects) in stereotype-congruent (e.g., boy-spatial) and stereotype-incongruent sorting conditions (e.g., girl-spatial). The assumption in this type of tests is that people with stronger stereotyped beliefs will respond faster to stereotype congruent than to stereotype incongruent conditions.

Explicit and implicit measures may assess different constructs. Children's answers on explicit measures are the result of conscious and deliberate thought. As a consequence, responding is sensitive to social desirability, in that children may inhibit their automatic thoughts and replace them with more socially accepted thoughts. Explicit beliefs may therefore reflect children's knowledge or awareness of common stereotypes, instead of their personal endorsement of the stereotype. In contrast, implicit measures assess unconscious and automatic responses that may reflect the personal acceptance of the stereotype (Devine, 1989; Cvencek et al., 2011). Importantly, this is not to say that implicit measures are accurate or real and explicit measures are not. Rather, it indicates that explicit and implicit measures are both important to consider when studying stereotypic beliefs (Nosek et al., 2011).

The majority of extant studies on gender beliefs in children makes use of explicit measures. For the spatial domain, there is evidence for the presence of explicit stereotypic gender beliefs in both boys and girls, although the results are somewhat inconsistent. Neuburger et al. (2015) measured 10-year-old children's gender beliefs on spatial ability by asking questions such as “Who is good at solving mental rotation tasks?,” and “Who is good at imagining spatially?.” Children answered on a five-point scale (i.e., only girls—more girls than boys—as many girls as boys—more boys than girls—only boys). The results showed that both boys and girls considered mental rotation as typically male. Spatial imagery was considered typically male by the boys, but gender-neutral by the girls. In contrast, in another study with 10-year-olds, the girls did not show a male stereotype for spatial abilities, such as mental rotation, spatial orientation and line reflection. However, the boys and girls in this study agreed that building and construction activities were more typical for boys than for girls (Ruthsatz et al., 2012).

Studies with implicit measures have not yet been performed for spatial ability, but there are some studies of mathematics in children. The study of Ambady et al. (2001) for example investigated the presence of the implicit stereotype that “math is for boys” by telling children a story about a student that was especially good at math. The sex of the student was never mentioned. The children had to repeat the story. Boys were more likely to use the word “he” when referring to the student (i.e., congruent with the stereotype), while the girls were as likely to identify the student as boy or girl. Cvencek et al. (2011) measured the presence of implicit sex stereotypic beliefs on mathematics with a child version of the Implicit Association Test (IAT, Greenwald et al., 1998). The results of this study showed that both boys and girls in elementary school had stronger associations of math with boys than with girls. Interestingly, only a weak correlation (i.e., *r* = 0.14) between the explicit and implicit measures was found in these children (Cvencek et al., 2011), suggesting that the explicit and implicit tasks indeed assessed different aspects of gender beliefs. Together, these studies show evidence for the presence of explicit sex stereotypic beliefs on spatial ability in 10-year-old boys and girls, with boys being more male stereotyped than girls. Studies with implicit measures have not yet been performed, but from studies on mathematics it seems reasonable to assume that these stereotypic beliefs are also present at an implicit level.

### Effects of gender beliefs on spatial performance

The short-term effects of gender beliefs on test performance have been studied extensively, especially in the domain of mathematics. Generally, it is assumed that positive information about the ability of the own sex promotes someone's confidence, self-esteem and self-efficacy, leading to performance increases (Shih and Pittinsky, 1999). On the other hand, negative beliefs about the ability of the own sex may cause psychological feelings of stress and lower self-confidence, leading to performance decreases–(Muzzatti and Agnoli, 2007; Nguyen and Ryan, 2008; Flore and Wicherts, 2015). It is argued that test performance is disrupted since the negative thoughts and worries occupy the capacity of working memory (Beilock et al., 2007; Schmader et al., 2008), required for fast and accurate test performance.

Whether gender beliefs also have short-term effects on spatial test performance, was firstly investigated in adults. Within a pretest—instruction—posttest design, Moè and Pazzaglia (2006) gave their adult participants one of the following three types of instruction: “men are better,” “women are better,” and neutral (no reference to sex differences). Women increased their performance in the “women better” condition, and decreased their performance in the “men better” condition. However, women who did not believe in men's superiority in spatial tasks *a priori*, as measured by a questionnaire, showed no decrease in performance. Having the idea that there are no sex differences in spatial ability was protective against instructions suggesting sex differences. Men in the “men better” condition had a significant increase in accuracy from pre- to posttest, and those in the “women better” condition showed a significant decrease. Men who a priori believed that men are much better in spatial ability than women, showed stronger effects of the “men better” instruction than men who believed a priori that there were few or no differences. Positive effects of instructions emphasizing the superiority of the own gender were replicated in later studies with adults (Moè, 2009; Heil et al., 2012).

Recently, two studies investigated the short-term effects of activating gender beliefs in 10-year-old children. The results are inconsistent. In contrast to findings with adults, Titze et al. (2010b) found no effects of instructing gender beliefs on mental rotation performance. Children's performance did not decrease or increase as a function of instruction: boys always outperformed girls; girls not even outperformed their same-sex counterparts given the “girls better” instruction. Neuburger et al. (2012), however, did find effects of experimentally manipulating gender beliefs. With a comparable pretest- instruction—posttest design they observed improvement in girls and deterioration in boys in the “girls better” and gender-neutral condition. In the “boys better” condition, boys and girls did not change their performance after instruction.

Taken together, studies in adults have found positive effects on spatial ability for manipulations in which the own gender was instructed to be superior, and negative effects for manipulations in which the other gender was instructed to be superior. Pre-existing gender beliefs on spatial ability interacted with the effect of the experimental manipulations. In children, the two studies performed in 10-year-olds showed inconsistent findings. One study found no effects of the manipulations at all, the other found negative effects for boys and positive effects for girls in the “girls better” instruction. These studies did not control for the effects of pre-existing gender beliefs. All in all, there is no consistent evidence for the claim that gender beliefs have direct effects on spatial test performance in late childhood.

### The current study

The overarching aim of the current study was to investigate whether gender beliefs may provide an explanation for the observed sex differences in spatial performance in late childhood. The study was comprised of two parts. In the first part we investigated whether 10- and 12-year-old children were characterized by explicit and implicit stereotypic gender beliefs on spatial ability (i.e., “spatial is for boys”) and we examined possible sex and age differences in these beliefs. The explicit gender beliefs were measured by a self-report questionnaire, assessing the degree to which children associated spatial activities with either boys, girls or both sexes. The implicit gender beliefs were measured by a child IAT (based on Cvencek et al., 2011), assessing children's implicit associations between “boy” and “spatial.” On basis of previous research, we expected boys to have stronger explicit and implicit stereotypic gender beliefs regarding spatial abilities and activities (i.e., spatial is for boys) than girls (Ruthsatz et al., 2012; Neuburger et al., 2015). In addition, we expected children's gender beliefs to be stronger male stereotyped in ten- than in 12-year-olds (Muzzatti and Agnoli, 2007).

In the second part of this paper we investigated, in the same group of children, whether activating gender beliefs has short-term effects on spatial performance. We examined differences between three instructions (i.e., boys are better, girls are better, no differences) in a pretest—instruction—posttest design. Taking into account that sex differences vary by type of spatial test (Voyer et al., 1995), we administered two well-known spatial tests, one showing sex differences (i.e., the Mental Rotation Test (Peters et al., 1995), the other showing no sex differences (i.e., the Paper Folding Test, Ekstrom et al., 1976). The effects of gender beliefs on spatial abilities other than mental rotation have not been investigated before (Reilly and Neumann, 2013). Previous studies on the effects of gender beliefs in children were only performed in 10-year-olds, and showed inconsistent results. We included 10-year-olds to get a more complete view on the effects in this age group, and 12-year-olds to examine whether the effects of gender beliefs are different in older children. With age, sex differences in mental rotation become larger (e.g., Geiser et al., 2008), and children's awareness about stereotypes increases (McKown and Weinstein, 2003). Therefore, we hypothesized that gender beliefs would have stronger effects on spatial ability in 12- than 10-year-olds. We extend previous studies by controlling for individual differences in pre-existing gender beliefs on spatial ability. We used the implicit measure, as previous studies found that implicit, and not explicit, gender beliefs were a significant indicator of sex differences in spatial performance at a national level (Nosek et al., 2009). In addition, we controlled for individual differences in spatial experience, as the literature provides clear evidence that sex differences in spatial ability relate to sex differences in experiences with spatial tasks and materials (e.g., Baenninger and Newcombe, 1995; Jirout and Newcombe, 2015; Moè, 2016). In line with previous studies in adults (Moè and Pazzaglia, 2006; Moè, 2009; Heil et al., 2012) and with the study of Neuburger et al. in children (2012), we expected positive effects of instructing gender superiority on the two types of spatial ability. In the “boys better” condition, we expected improvement in the boys. In the “girls better” and gender-neutral condition we expected improvement in the girls.

## Methods

### Participants

In this study, 237 children (47% boys, 53% girls) between 7 and 13-years participated; 134 children were from grade 4 (*M* age = 9.92, *SD* = 0.56) and 103 children were from grade 6 (*M* age = 12.00, *SD* = 0.44). Children were recruited from six regular elementary schools from different regions in the Netherlands. Children were from 15 different classes and were divided (class by class) into one of three groups of experimental manipulation: boys better (*n* = 81), girls better (*n* = 83), and no gender difference (*n* = 73). The distribution of children over the three conditions is presented in Table 1. The children in the three experimental conditions did not differ in their scores on the Raven SPM (Raven et al., 2000), a measure for non-verbal intelligence, *F*_(2, 233)_ = 1.31, *p* = 0.27 (Table 1). Parents gave written informed consent. The Ethical Committee of the Faculty of Behavioral and Movement Sciences of Vrije Universiteit Amsterdam approved the research protocol.

**Table 1:** **Number of Children and Raven Scores in the Three Experimental Conditions**.

	**Boys better**	**Girls better**	**No differences**	**Total**
Grade 4 (girls)	45 (27)	44 (22)	45 (27)	134 (76)
Grade 6 (girls)	36 (15)	39 (18)	28 (16)	103 (49)
Total (girls)	81 (42)	83 (40)	73 (43)	237 (125)
Raven SPM (max score 48)	34.10 (5.78)	32.82 (5.29)	32.90 (5.70)	33.28 (5.60)

### Materials

#### Mental rotation

The effects of the gender belief instruction on spatial ability were tested in a pretest-instruction-posttest design. To measure mental rotation ability we administered the Revised Vandenberg and Kuse Mental Rotations Test (Peters et al., 1995). As the items of this test do not increase in difficulty, we split the 24 items of the test in a pretest (items 1–12) and a posttest (items 13–24). The items consisted of three-dimensional cube constructions. Each item consisted of a target object on the left and four objects on the right. The participant was required to determine which two (out of four) stimuli were rotations, and not mirror versions, of the target figure. To avoid the risk of test instructions being too difficult for the youngest age group (Hoyek et al., 2012), we used a physical example item to show that all figures of a problem had the same constellation of cubes, but were rotated around the vertical axis. The time limit was 4 min per part of 12 items. One point was given if the child identified *both* correct answers, with a maximum score of 12.

#### Paper folding

The Paper Folding Test (PFT, Ekstrom et al., 1976) required the children to predict from two, three, or four pictures how a square piece of paper would look after it had been folded, a hole had been punched in it, and it had been unfolded again. The test consisted of 20 problems in total, which we again divided in a pretest (item 1–10) and a posttest (item 11–20). We used a model paper for the instructions. The time limit was 3 min per part of 10 problems. The child got one point for each correct answer. The variable of interest was the total number of correct answers, with a maximum score of 10.

#### Non-verbal intelligence

We administered the Raven Standard Progressive Matrices (Raven et al., 2000) as a measure of non-verbal reasoning ability. Children were instructed to solve as many of the 48 items (only sections A–D, not E) in 10 min. The variable of interest was the total number of correct answers, with a maximum score of 48.

#### Spatial experience

We presented the children a paper-and-pencil questionnaire, that was adapted from the Spatial Activity Questionnaire (Dearing et al., 2012). We selected 18 spatial activities that we considered suitable for 10- and 12-year-old Dutch children. Example items are “Playing with paper airplanes,” “Climbing trees,” and “Using tools such as a hammer or screwdriver to make things.” We added “Doing ball sports, such as soccer, tennis, hockey” and “Doing sports without a ball, such as judo, dancing, or cycling” as 19th and 20th item (see Attachment A for the complete list of items). We asked the children to indicate on a three-point Likert-scale (“never” [0], “sometimes” [1], “often” [2]) how often they participated in each spatial activity in their free time. The variable of interest was the mean of these 20 items, with a maximum of 2, and with higher scores indicating greater participation in spatial activities. The internal consistency of this questionnaire was good with a Cronbach's alpha of 0.80 for the boys and 0.81 for the girls.

#### Explicit gender beliefs on spatial ability

The measure for explicit gender beliefs was based on the 20 activities of the spatial experience questionnaire (see paragraph Spatial Experience). The measure comprised two parts. In the first part, the activities were presented in a randomly different order and we asked the children to answer for each activity the following question: “What do you think, is this activity more appropriate for boys [1] or for girls [–1]? Or for both? [0]” (Cronbach's α = 0.72). In the second part, the activities were shuffled again, and now the children were asked: “Who do you think are better skilled in this activity? Boys or girls? Or are boys as good as girls?” (Cronbach's α = 0.73). For both part 1 and 2 we computed the mean score on the 20 activities, with scores ranging between −1 and 1. Positive scores indicated stronger association of the spatial activities with boys, and negative scores stronger association of the spatial activities with girls.

#### Implicit gender beliefs on spatial ability

To measure children's implicit gender beliefs on spatial ability, we adapted the Child Math IAT for elementary school children (Cvencek et al., 2011). This computerized categorization task measures the relative strengths of associations between two concepts (i.e., sex and school subject), each presented in two categories (i.e., boy names vs. girl names and math words vs. language words). Where the Child Math IAT of Cvencek et al. (2011) investigated implicit associations between boy and mathematics, we investigated associations between boy and spatial ability.

The IAT started with two single discrimination “practice” blocks, each consisting of 16 trials. First, children practiced sorting *boy* and *girl* names. They responded to Dutch boy names (i.e., Bram, Tim, Bart, Stijn) by pressing a response button on the left side of the keyboard (the “A” key marked by a yellow sticker) and to girl names (i.e., Emma, Sanne, Lotte, Lieke) by pressing a response button on the right side (the “L” key, marked by a green sticker). Second, children practiced sorting *spatial* words (i.e., numbers, sums, constructing, measuring) and *language* words (letters, sentences, reading, writing), using the same response buttons. As we were not sure that all children were familiar with the concept “spatial,” we labeled this category “Math and technology,” which refers to familiar “spatial” subjects in their daily school program. After these two practice blocks, children completed two combined discrimination blocks, in which all four categories were used. During the combined blocks, each consisting of 24 trials, two of the four categories were mapped onto the same response key. In one block, *spatial* words and *boy* names shared one response key, with *language* words and *girl* names sharing the other (stereotype congruent). The other block was stereotype incongruent, with *spatial* paired to *girl* and *language* paired to *boy*. The IAT score (Greenwald et al., 2003) was calculated by comparing the response speed of congruent and incongruent blocks. Children with stronger stereotypic gender beliefs (i.e., boy = spatial), were expected to respond faster to the stereotype congruent than to the stereotype incongruent condition of the task (Cvencek et al., 2011).

The task was developed and administered in Inquisit version 4.0.4.0^1^. In contrast to the Child IAT of Cvencek et al. (2011), the stimuli words were only written, and not spoken, as we assumed that the children were able to read them in the time frame used. Each block started with a wait trial (blank screen) for 2000 ms. Each stimulus trial started with a small asterisk in the middle of the screen to fixate children's attention. After 300 ms the stimulus word appeared. There was no limit to the response time. That is, children always had to choose between the left or right response button to proceed to the next trial. Errors were indicated to the children with the word “incorrect” appearing in red in the middle of the screen for 1000 ms. Incorrect trials were not repeated. After a correct answer, testing proceeded to the next trial without information on correct or incorrect. The intertrial interval was 500 ms. The order of the congruent and incongruent blocks was counterbalanced. We recorded accuracy and response latency per trial. IAT scores were computed using the improved scoring algorithm of Greenwald et al. (2003). For both sexes we subtracted the stereotype congruent block (spatial = boy) from the stereotype incongruent block (spatial = girl). Positive scores indicated stronger association of spatial with boys, and negative scores indicated stronger association of spatial with girls.

In addition to this gender belief IAT, measuring the associations between sex and spatial ability, we administered a “gender identity IAT” and a “spatial self-concept IAT” (as done by Cvencek et al., 2011 for math). As these two IATs were not related to the research questions of the current paper, no data about these measures are discussed. We did not use IAT data of participants with (a) 10% or more of their responses faster than 300 ms, (b) an error rate of 35% or greater in at least one of the three IATs, or (c) an average response latency 3 *SD* above the mean response latency for the whole sample in at least one of the three IATs (Greenwald et al., 2003). These criteria excluded 14 of the 237 participants (5.9%). These were seven children from grade 4 (5 boys, 2 girls) and seven children from grade 6 (3 boys, 4 girls).

### Procedure

Children were tested during regular class time in mixed-sex groups of 15–25 children by female experimenters. In the first classical test session, children were informed about the research protocol (i.e., nothing was said about sex differences) and they completed the explicit gender belief questionnaire (15 min) and the Raven SPM (10 min). During the same day, we administered the children, in groups of three of four children, in a quiet room in the school, the implicit gender belief IAT at individual laptop computers. The gender identity IAT and spatial self-concept IAT were counterbalanced in the first and third position, with the spatial-gender stereotype IAT always administered in the second position. Two weeks later, the experimental manipulation took place in a classical session of about 50 min. Each child received a test booklet and sat alone at its own desk. First, children had to complete the pretest of the MRT (4 min) and PFT (3 min). The experimenters read out aloud the instructions from the test booklet. Both tasks started with a practice item. After the pretests, we presented the experimental manipulation (based on Titze et al., 2010a), which we read out aloud to the children, and was printed in their booklets. The children in the “boys better” condition were told: “This is important information. There is something we have to tell you about these tasks. Many children in the Netherlands have already completed these tasks. We have counted the number of correct answers. Boys always performed much better than girls on these tasks. Boys always had more correct answers than girls. Now we want to check whether in this class the boys are better too.” The children in the “girls better” condition were told that the girls were better than the boys. The children in the gender-neutral condition were told: “Boys were as good as girls in these tasks. Boys always had as many answers correct as girls. Now we want to check whether in this class the boys are as good as girls too.” After the manipulation, the children had to complete the posttests of the MRT and PFT. Afterwards, we debriefed the children on the goals of the study and answered all their questions.

## Results

The analyses consisted of two steps. In the first step, we investigated the presence of explicit and implicit stereotypic gender beliefs on spatial ability, and sex and grade differences in these beliefs. In the second step, we examined the effects of experimentally manipulating children's gender beliefs (i.e., boys better, girls better, or no differences) on boys' and girls' spatial test performance (i.e., mental rotation and paper folding), while controlling for individual differences in pre-existing gender beliefs and spatial experience.

### Explicit and implicit gender beliefs on spatial ability

#### Explicit gender beliefs

**Figure 1:**
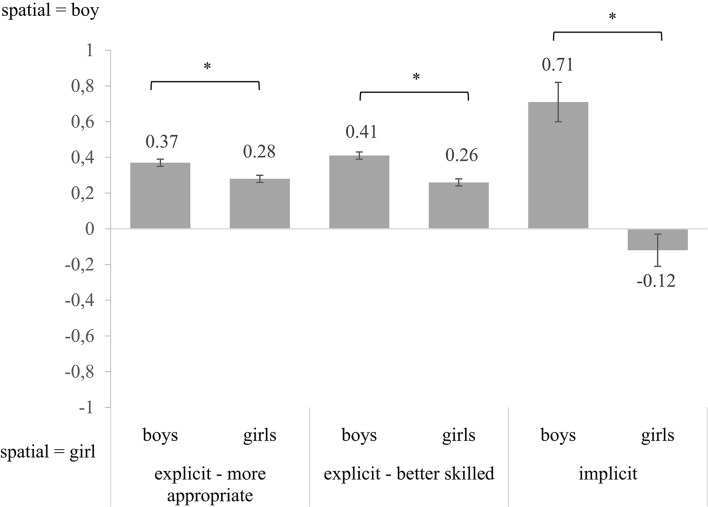
**Sex differences on the explicit and implicit gender beliefs**. The error bars represent the standard errors of the mean. For all three measures the difference between boys and girls was significant. ^*^Refers to significant difference at *p* < 0.05.

First, we tested the hypothesis that 10- and 12-year-old children already had explicit stereotypic beliefs on sex differences in the spatial domain (i.e., the questionnaires with boy-girl statements). In addition, we examined whether these explicit stereotypic beliefs were stronger in the boys and in the older children (see Table 2 and Figure 1). An ANOVA on part two of the questionnaire (“What do you think, are these activities more appropriate for boys or girls? Or for both?”) showed a main effect of sex, *F*_(1, 229)_ = 19.21, *p* < 0.001, η_*p*_^2^ = 0.08 (medium effect), but no main effect of grade, *F*_(1, 229)_ = 0.07, *p* = 0.79, and no interaction effect, *F*_(1, 229)_ = 2.49, *p* = 0.12. Both boys and girls considered the spatial activities more appropriate for boys than girls (i.e., for both sexes scores were positive and significantly different from 0, *p*s < 0.001). Boys had stronger associations between spatial and boy than the girls. For part three (“Who do you think are better in these activities? Boys or girls? Or are boys as good as girls?”) we found a main effect of sex, *F*_(1, 228)_ = 39.61, *p* < 0.001, η_*p*_^2^ = 0.15. Again there was no main effect of grade, *F*_(1, 228)_ = 0.61, *p* = 0.44, and no interaction effect of sex and grade, *F*_(1, 228)_ = 0.69, *p* = 0.41. Both boys and girls considered boys more skilled in spatial activities than girls (i.e., for both sexes scores were positive and significantly different from 0, *p*s < 0.001). Boys had stronger associations between boy and spatial than girls. The two parts of the questionnaire were strongly interrelated in both the boys (*r* = 0.64, *p* < 0.001) and the girls (*r* = 0.57, *p* < 0.001).

**Table 2:** **Scores on the Explicit Gender Belief Questionnaires (***N*** = 237) and the Implicit Gender Belief IAT (***N*** = 223), Separately for Sex and Age**.

	**Grade**	**Total (*M*, *SD*)**	**Boys (*M*, *SD*)**	**Girls (*M*, *SD*)**	
Explicit–more appropriate	4	0.31 (0.17)^*^	0.38 (0.17)^*^	0.26 (0.14)^*^	
	6	0.33 (0.15)^*^	0.36 (0.15)^*^	0.30 (0.13)^*^	
	Total	0.32 (0.16)^*^	0.37 (0.16)^*^	0.28 (0.14)^*^	Boys > Girls
Explicit—better skilled	4	0.32 (0.20)^*^	0.41 (0.16)^*^	0.25 (0.20)^*^	
	6	0.35 (0.18)^*^	0.41 (0.17)^*^	0.29 (0.16)^*^	
	Total	0.34 (0.19)^*^	0.41 (0.17)^*^	0.26 (0.18)^*^	Boys > Girls
Implicit	4	0.30 (1.12)^*^	0.74 (1.23)^*^	−0.03 (0.91)	
	6	0.24 (1.23)	0.68 (1.13)^*^	−0.24 (1.17)	
	Total	0.27 (1.17)^*^	0.71 (1.18)^*^	−0.12 (1.02)	Boys > Girls

*Positive scores represent stronger associations of spatial with boy. Negative scores represent stronger associations of spatial with girl. Values with an asterisk represent scores that are significantly different from zero. Explicit and implicit scores cannot be compared with each other, as these are measured with different tasks. Interpretation of effect size of the implicit measure: 0.20 = small, 0.50 = medium, 0.80 = large (Greenwald et al., 1998)*.

#### Implicit gender beliefs

Next, we tested the same hypotheses for the implicit gender beliefs (i.e., the computerized IAT). The ANOVA showed a main effect of sex, *F*_(1, 230)_ = 34.07, *p* < 0.001, η_*p*_^2^ = 0.13, but no main effect of grade, *F*_(1, 230)_ = 0.86, *p* = 0.35, and no interaction effect, *F*_(1, 230)_ = 0.28, *p* = 0.60 (see Table 2 and Figure 1). Boys strongly associated spatial activities and abilities with boys (i.e., positive IAT score, significantly different from 0, *p* < 0.001), while girls associated spatial abilities as strongly with boys as with girls (i.e., score not significantly different from 0, *p* = 0.21).

The correlations between the explicit and implicit gender belief measures were weak for part two of the questionnaire (*r* = 0.07, *p* = 0.31) and small for part three of the questionnaire and (*r* = 0.20, *p* = 0.002), suggesting that the implicit and explicit tasks measured different aspects of gender beliefs.

Taken together, at the explicit level, both sexes believed that boys are more appropriate for and better skilled in spatial activities than girls, with these beliefs being stronger male stereotyped in boys than in girls. At the implicit level, boys strongly associated the spatial domain with boys, while girls were gender-neutral. There were no differences in the explicit and implicit gender beliefs between children from grade 4 and 6 (i.e., between the 10- and 12-year-olds) and no interactions between sex and grade. These findings confirm the hypothesis that sex stereotypic beliefs on spatial ability are already present in 10- and 12-year-olds. These beliefs are stronger in boys than in girls, especially at the implicit level.

### Effects of gender beliefs on spatial ability

In the second step of the analyses, we tested the hypothesis that instructing boys and girls that their own sex is superior in the spatial domain would have positive effects on their spatial test performance, while controlling for individual differences in pre-existing gender beliefs and spatial experience. Preliminary to the main analysis, we examined sex and grade differences on the pretest of the mental rotation and paper folding task, we examined sex and grade differences in spatial experience, and we computed correlations between the pre-existing gender beliefs and spatial performance.

#### Sex and grade differences in spatial ability

We examined differences between children on the pretest of the MRT (i.e., mental rotation) and PFT (i.e., paper folding; Table 3). An univariate analysis of variance with scores on the MRT as dependent factor and sex (boys, girls) and grade (4, 6) as independent factors showed a main effect of sex, *F*_(1, 233)_ = 8.56, *p* = 0.004, η_*p*_^2^ = 0.04 (small effect) and grade, *F*_(1, 233)_ = 22.80, *p* < 0.001, η_*p*_^2^ = 0.09 (medium effect), but no interaction effect of sex and grade, *F*_(1, 233)_ = 0.60, *p* = 0.44. Boys outperformed girls and grade 6 outperformed grade 4. For the PFT, the ANOVA showed a large main effect of grade, *F*_(1, 233)_ = 38.08, *p* < 0.001, η_*p*_^2^ = 0.14, but no main effect of sex, *F*_(1, 233)_ = 0.10, *p* = 0.75 and no interaction effect of sex and grade, *F*_(1, 233)_ = 0.01, *p* = 0.91. There were no sex differences in paper folding. Grade 6 outperformed grade 4.

**Table 3:** **Pretest scores on the Mental Rotation Test (MRT) and Paper Folding Test (PFT), separately for sex and grade**.

	**Grade**	**Total (*M*, *SD*)**	**Boys (*M*, *SD*)**	**Girls (*M*, *SD*)**	
MRT	4	3.91 (2.28)	4.33 (2.48)	3.59 (2.08)	
	6	5.62 (3.03)	6.22 (3.21)	4.96 (2.69)	
	Total	4.65 (2.76)	5.24 (3.00)	4.13 (2.42)	Boys > Girls, Grade 6 > 4
PFT	4	3.47 (1.62)	3.41 (1.71)	3.51 (1.55)	
	6	4.89 (1.92)	4.87 (2.08)	4.92 (1.75)	
	Total	4.09 (1.89)	4.12 (2.02)	4.06 (1.77)	Boys = Girls, Grade 6 > 4

#### Sex and grade differences in spatial experience

We examined differences between children in spatial experience. An univariate analysis of variance with scores on the spatial experience questionnaire as dependent factor and sex (boys, girls) and grade (4, 6) as independent factors showed no main effect of sex, *F*_(1, 229)_ = 1.06, *p* = 0.32, but a main effect of grade, *F*_(1, 229)_ = 26.08, *p* < 0.001, η_*p*_^2^ = 0.10 (medium effect). There was no interaction effect of sex and grade, *F*_(1, 229)_ = 0.05, *p* = 0.82. No differences in spatial experience between boys (*M* = 0.71*, SD* = 0.28) and girls (*M* = 0.69*, SD* = 0.26) were observed. Children from grade 4 (*M* = 0.78*, SD* = 0.24) participated more frequently in spatial activities than children from grade 6 (*M* = 0.61*, SD* = 0.28).

#### Correlations between pre-existing gender beliefs and spatial ability

We computed correlations between boys' and girls' pre-existing gender beliefs and their mental rotation and paper folding scores on the pretest, separately for grade 4 and 6. Boys' gender beliefs did not relate to their spatial test scores (all *r*s < 0.18, *p*s > 0.05). Also in the girls, most correlations between the gender beliefs and spatial test scores were non-significant, with two exceptions. First, there was a positive correlation between the explicit gender belief measure “better skilled” and the paper folding scores of the girls in grade 4 *(r* = 0.24, *p* = 0.04). Stronger explicit beliefs that boys are better skilled in the spatial domain were related to higher paper folding scores. Second, there was a negative correlation between the explicit gender belief measure “more appropriate” and the paper folding scores of the girls in grade 6 *(r* = −0.34, *p* = 0.02). Stronger explicit beliefs that boys are more appropriate for the spatial domain were related to lower paper folding scores.

#### Effects of the experimental manipulations

With repeated measures ANOVAs we investigated pretest-posttest differences between the three experimental conditions (i.e., boys better, girls better, no gender difference) and between the sexes (boys, girls) and grades (4, 6), separately for mental rotation and paper folding (Table 4). The implicit gender belief and spatial experience measure were added as covariates in the analyses.

**Table 4:** **Mental rotation and paper folding scores before and after instruction, separately for sex and grade**.

	**Boys**			**Girls**		
	**Pretest**	**Posttest**	***d***	**Pretest**	**Posttest**	***d***
**MRT**						
**GRADE 4**						
Boys are better	3.72 (1.87)	3.56 (1.98)	0.08	3.41 (2.31)	3.81 (2.24)	0.18
Girls are better	3.82 (1.87)	4.45 (2.58)	0.28	3.64 (2.04)	3.86 (2.21)	0.10
Gender-neutral	5.56 (3.24)	5.94 (3.19)	0.12	3.74 (1.93)	3.67 (1.73)	0.04
**GRADE 6**						
Boys are better	6.95 (3.23)	6.71 (3.09)	0.08	5.20 (2.78)	5.73 (3.11)	0.18
Girls are better	5.43 (3.27)	6.10 (3.52)	0.20	4.83 (2.62)	5.00 (1.91)	0.07
Gender-neutral	6.33 (3.03)	6.50 (3.29)	0.05	4.88 (2.83)	5.37 (2.85)	0.17
**PFT**						
**GRADE 4**						
Boys are better	3.00 (1.65)	3.39 (2.17)	0.20	3.85 (1.94)	4.07 (2.18)	0.11
Girls are better	3.32 (1.46)	3.82 (2.06)	0.28	3.23 (1.34)	4.23 (2.00)	0.59
Gender-neutral	3.94 (1.98)	3.89 (2.03)	0.02	3.41 (1.25)	4.11 (1.55)	0.50
**GRADE 6**						
Boys are better	5.48 (2.29)	5.76 (1.55)	0.14	5.60 (2.41)	5.60 (2.06)	0
Girls are better	4.24 (1.92)	4.95 (1.91)	0.37	4.39 (1.42)	5.05 (2.24)	0.35
Gender-neutral	4.92 (1.73)	4.58 (2.07)	0.18	4.87 (1.15)	5.75 (2.08)	0.52

*Cohen's d: 0.20 = small effect, 0.50 = medium effect, 0.80 = large effect. These scores are uncorrected for the covariate*.

##### Mental rotation

The repeated measures ANOVA with the pretest and posttest scores on mental rotation as within-subjects factor, condition, sex, and grade as between-subjects factors and the implicit gender belief and spatial experience measures as covariates revealed main effects of sex [*F*_(1, 216)_ = 9.26, *p* = 0.003, η_*p*_^2^ = 0.04], and grade [*F*_(1, 216)_ = 29.27, *p* < 0.001, η_*p*_^2^ = 0.10], but no main effects of time and condition. Boys outperformed girls and grade 6 outperformed grade 4, but there were no significant differences between the pretest and posttest and between the three experimental conditions. We did not find any significant interaction effect between the factors, indicating that there were no differences in pretest-posttest progress between the three experimental conditions and between the sexes and grades. In addition, pretest-posttest progress was not related to children's pre-existing gender beliefs and their spatial experience.

##### Paper folding

The repeated measures ANOVA with the pretest and posttest scores on paper folding as within-subjects factor, condition, sex, and grade as between-subjects factors and the implicit gender belief and spatial experience measures as covariates revealed a main effect of grade [*F*_(1, 216)_ = 40.63, *p* < 0.001, η_*p*_^2^ = 0.16], but no main effects of time, sex and condition. Grade 6 outperformed grade 4. There was no significant improvement from pretest to posttest and there were no significant differences between the sexes and between the three experimental conditions. We did not find any significant interaction effect between the factors, indicating that there were no differences in pretest-posttest progress between the three experimental conditions and between the sexes and grades. In addition, there were no effects of children's pre-existing gender beliefs and spatial experience on pretest-posttest progress.

Taken together, the hypothesis that positive information about the ability of the own sex would have positive effects on spatial test performance was not confirmed. We found no direct effects of the gender belief instructions on children's mental rotation and paper folding performance.

## Discussion

Spatial ability is stereotypically considered a male aptitude (Nash, 1979; Neuburger et al., 2013). Especially in late childhood, such stereotypic beliefs may provide an explanation for observed sex differences in spatial ability. Children of this age have developed awareness of stereotypes (McKown and Weinstein, 2003) and make important steps in the development of self-concepts of ability (Berk, 2013). However, the literature is inconclusive whether at this age (1) children (already) have stereotypic beliefs on sex differences in spatial ability; (2) there are short-term effects of gender beliefs on spatial test performance, as observed in studies with adults (e.g., Moè and Pazzaglia, 2006; Heil et al., 2012). We investigated these two topics in 10- and 12-year-old children, by examining with both explicit and implicit measures the presence of stereotypic gender beliefs on spatial ability and by examining the effects of experimentally manipulating children's beliefs about sex differences in spatial ability (i.e., instructing children that either boys are better, girls are better, or that there are no gender differences) on spatial performance.

The results showed that stereotypic beliefs on sex differences in the spatial domain were already present in 10- and 12-year old children. Boys had strong explicit (i.e., conscious) and implicit (i.e., unconscious) male stereotyped beliefs regarding spatial abilities (i.e., boys are superior to girls in the spatial domain). Girls agreed with this stereotype on the explicit measure, although they were less male stereotyped than boys. On the implicit measure however they showed gender-neutral beliefs about sex differences in spatial ability (i.e., no sex is superior). This incongruence indicates that girls, in accordance with social desirable standards, answered that “spatial is for boys” on the self-report questionnaire, but that they did not personally endorse this stereotype. The finding of girls being more egalitarian than boys is in line with previous studies with explicit measures in the spatial domain (Ruthsatz et al., 2012; Neuburger et al., 2015) and in mathematics (Muzzatti and Agnoli, 2007). The incongruence between explicit and implicit measures of girls' gender beliefs underlines the importance of further examining the interrelations and developmental sequence of these two types of beliefs. We did not observe differences between the 10- and 12-year-old children, suggesting that children's ideas about male superiority in the spatial domain do not change in this age period.

In general, children's explicit and implicit beliefs about sex differences in the spatial domain did not relate to their spatial performance on the pretest. Probably there was no relation because most children were completely unfamiliar to the mental rotation and paper folding task at pretesting. The majority of girls was therefore not aware of the fact that they were taking tests on which boys are (assumed to be) better. Therefore, in advance, the test may not have evoked any feelings of stress or lowered self-confidence. This may be different for mathematics, since children have daily experience with this kind of tasks and, as a consequence, may have strongly established beliefs about the ability of their own sex (e.g., Muzzatti and Agnoli, 2007).

In the second part of the analyses, we investigated the short-term effects of explicitly instructing children that either boys are better on spatial tasks, girls are better, or there are no sex differences, while accounting for individual differences in pre-existing implicit stereotypes. First, children were administered a mental rotation and paper folding pretest. In line with the literature (Voyer et al., 1995), boys outperformed girls on the mental rotation test, but not on the paper folding task. These findings support the growing body of evidence that sex differences in mental rotation emerge before adolescence, probably around 10-years of age (e.g., Johnson and Meade, 1987; Titze et al., 2010a; Neuburger et al., 2011; Hoyek et al., 2012).

The instructions (i.e., boys are better, girls are better, no sex differences) were given in a pretest-instruction-posttest experiment. We found no differences in progress between the pretest and posttest between the three instructions, suggesting that in 10- and 12-year-olds there were no direct effects of induced gender beliefs on spatial performance. These findings replicate the findings of Titze et al. (2010b), showing in a similar study no effect of induced gender beliefs on 10-year-old children's spatial performance, but are in contrast with the study of Neuburger et al. (2012), showing positive effects in girls and negative effects in boys of the “girls are better” instruction (same procedure, but letter rotation task as posttest).

The absence of manipulation effects may be explained in different ways. First, children may not have identified themselves with the gender beliefs we tried to activate. Results of the implicit IAT showed that there were sex differences in how strongly boys and girls associated children of their own sex with the spatial domain, but possibly there were no sex differences in how strongly the children associated *themselves* with the spatial domain. It is possible that the children regarded the induced gender beliefs as true for other boys or girls, but considered themselves as a member of a subgroup for which the information did not apply, a process called stereotype stratification (Steele, 2003). The last sentence of our experimental instruction (i.e., “Now we want to check whether in this class the boys/girls are better too.”) may have strengthened this stratification effect (e.g., “The stereotype may apply to other girls, but not to girls in my class”). Second, the explicit priming of gender beliefs to mixed-sex groups in the natural setting of the classroom may have stimulated a “competition between the sexes,” resulting in increased effort and performance boosts in both sexes, regardless of which belief was activated. In line with this hypothesis, we found for none of the sexes and for none of the instructions *decreases* in spatial performance after instruction. Related to this, children may not have considered the induced sex differences as fixed and immutable (e.g., resulting from genetic differences between boys and girls), but instead, they may have attributed success on the spatial tasks to personal effort. Previous research showed positive effects of such effort attributions on spatial performance (Moè and Pazzaglia, 2010). Fourth, we measured direct and very short-term effects of instruction on spatial test performance. The effects of gender belief instructions on spatial performance may be stronger when the instructions are repeated multiple times, when children get more time to think about the instructed sex differences, or when children are asked to reflect on the instructions, for example by repeating them in their own words (e.g., Ambady et al., 2001).

Further, studies are necessary to clarify the direct and indirect effects of gender beliefs on spatial performance in children. First, studies might investigate, ideally with longitudinal studies, developmental changes in explicit and implicit stereotypical beliefs during the course of elementary school (as done for math by Cvencek et al., 2011). Relations between these beliefs and children's spatial self-confidence and interest for the spatial domain are important topics of investigation. Second, studies might examine why manipulating gender beliefs seems to have strong direct effects on spatial performance in adults (Moè and Pazzaglia, 2006; Moè, 2009; Heil et al., 2012), but not in children (this study, Titze et al., 2010b). Previous studies finding effects of gender beliefs in adults were administered individually (Heil et al., 2012) and in same-sex groups (Moè, 2009). To investigate the developmental determinants of stereotypic effects, different age groups should be included in a mixed-sex study design. Levels of self-confidence and working memory could be measured before and after instruction. Self-confidence and working memory have combined effects on spatial performance: confidence reduces stress and the need to suppress negative thoughts, thereby liberating working memory capacity for use on mental rotations (Schmader et al., 2008).

In conclusion, this study demonstrated that 10- and 12-year-old boys had both explicitly and implicitly the belief that their ability and performance in the spatial domain is superior to girls. Girls agreed with this stereotype when asked explicitly, but implicitly, they endorsed gender-neutral beliefs. There were no relations between these internalized gender beliefs and children's spatial test performance. In addition, test performance was not directly affected by explicit instructions that either boys were better, girls were better or there were no sex differences on the given spatial tests.

Although children's gender beliefs did not have short-term effects on spatial performance yet, we should watch for the long-term effects. For example, studies in adults showed significant associations between implicit stereotypic beliefs and women's plans to pursue science-oriented academic programs and careers (e.g., Lane et al., 2012). The stereotypic gender beliefs that we found to be present in 10- and 12-year-olds may affect boys' and girls' feelings of confidence and comfort in gathering relevant spatial experiences. These differences in spatial experience may result in performance differences between the sexes and ultimately to different educational and occupational choices (Bussey and Bandura, 1999). We therefore encourage teachers and caregivers to promote spatial confidence in girls by engaging them in a variety of spatial activities inside and outside school. The period around 10-years of age may be an important time window for such initiatives, as we observed that girls of this age were aware of the common belief that men are better in the spatial domain than women, but did not have personally endorsed this belief yet.

## Author contributions

KV, NV, and MH contributed to the development of the study hypotheses. KV and NV contributed to the study design. KV performed the data analysis and drafted the manuscript with input from NV. Critical revisions were contributed by MH and JJ. All authors discussed the results, implications, and literature, and approved the final version of the manuscript for submission.

### Conflict of interest statement

The authors declare that the research was conducted in the absence of any commercial or financial relationships that could be construed as a potential conflict of interest.

## Footnotes

^1^Inquisit (Version 4.0.4.0). [Computer Software]. Seattle, WA: Millisecond Software.

## Appendix A

### Items of the spatial activity questionnaire

**Table:**

1	Playing with toy soldiers, action figures, cars or trains
2	Folding paper airplanes
3	Doing arts and crafts projects (such as making jewelry, or using play dough/clay)
4	Coloring, painting, or drawing free hand
5	Using tools (such as hammer or screwdriver) to make things
6	Taking things apart to see how they work
7	Building a city with toy buildings, train tracks or building blocks
8	Searching for plants, bugs, or animals outdoors
9	Racing with toy animals or cars
10	Building with construction toys (such as building blocks, Legos)
11	Playing with puzzles
12	Drawing maps
13	Drawing houses, forts, castles, or other buildings
14	Playing in parks or green spaces
15	Using model building kits
16	Climbing trees
17	Playing with flying toys (such as kites, paper planes)
18	Building dams, forts, tree houses, or snow tunnels
19	Doing ball sports (such as football, tennis, hockey)
20	Doing sports without a ball (such as judo, dancing or cycling)
